# Effect of ultrasound combined with TGase-type glycation on the structure, physicochemical, and functional properties of casein hydrolysate

**DOI:** 10.1016/j.ultsonch.2025.107323

**Published:** 2025-03-23

**Authors:** Huimin Wang, Yujun Jiang, Jia Shi

**Affiliations:** a Department of Food Science, Key Laboratory of Dairy Science, Ministry of Education, Northeast Agricultural University, Harbin 150030, PR China; b Key Laboratory of Infant Formula Food, State Administration for Market Regulation, Harbin 150030, PR China

**Keywords:** Ultrasound treatment, Enzymatic hydrolysate, Glycation, TGase, Properties

## Abstract

This study investigated the effects of transglutaminase (TGase)-type glycation combined with ultrasound treatment on the structure, physicochemical properties, and functional properties of casein hydrolysate (CH). The results showed that TGase-type glycation and ultrasound treatment changed the secondary structure and reduced the fluorescence intensity of CH. Structural analysis revealed the intermolecular covalent interactions between oligochitosan and CH, confirming the occurrence of TGase-type glycation. The microstructure indicated that after 200 W sonication treatment, the structure of glycated CH was expanded and the molecular flexibility was enhanced. In addition, glycated CH treated with ultrasound treatment exhibited superior solubility, foaming capacity, antioxidant activity, and thermal stability. This study provides new insights into the combination of TGase-type glycation and ultrasound treatment, which may improve the function of casein and further increase its application in the food industry.

## Graphical abstract

**Figure:**
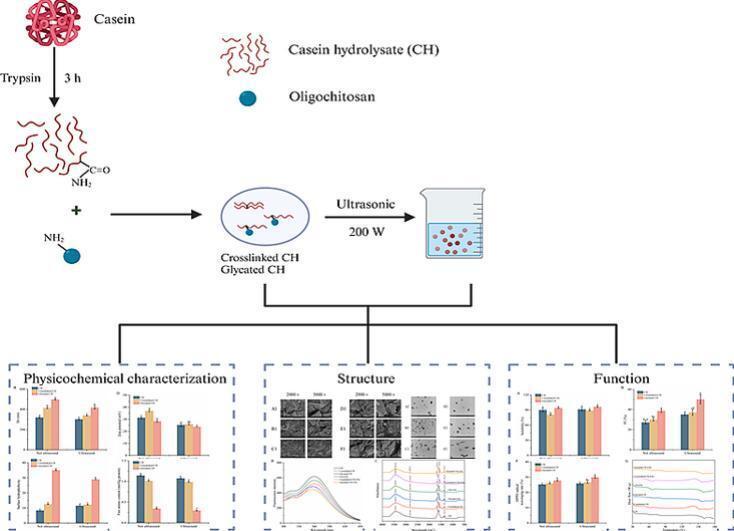

## 1 Introduction

Casein is widely used as a functional ingredient in the food industry due to its high nutritional value, substantial health benefits, ease of production, and affordable price [1]. It can also be used as additives to improve the technical and functional properties of different foods, such as viscosity, structure, gel texture, emulsifying and foaming properties. Nonetheless, the utilization of casein is limited by insufficient solubility, sensitivity to pH fluctuations, and isoelectric point precipitation. Therefore, modification strategies are required to enhance stability and functional characteristics of casein. Casein can become more soluble through hydrolysis, which can alter the structure and enhance its functional characteristics. During the process of casein hydrolysis, part of hydrophobic sites of casein are gradually exposed, and the resulting casein hydrolysate is a mixture of proteins, peptides, and amino acids. Casein is rich in antioxidant amino acids such as Tyr, Trp, and Met. After appropriate breakdown by proteases, it can generate antioxidant peptides with strong free radical scavenging ability [2]. It also exhibited stronger antioxidant activity than the parent protein. In addition, they can prevent oxidative stress in the plasma from producing excessive free radicals, thereby reducing the risk of aging [3]. However, the hydrolysis of casein produced large amounts of small peptides, which made some of its properties unstable.

To certain extent, the binding of polysaccharides with protein hydrolysates can enhance their functional properties. Polysaccharides and protein hydrolysates are bound by covalent or non-covalent binding (such as electrostatic binding) [4]. For the electrostatic interaction between proteins and polysaccharides, the combination products will dissociate due to the changes in ionic strength, pH value, and charge distribution [5]. Covalent binding with protein hydrolysates usually occurs in alkaline environments or in the presence of oxidases [6]. Polysaccharides and protein hydrolysates are covalently linked to form conjugates with permanent binding properties. Currently, the Maillard reaction is the most commonly used method in covalent reactions. However, the Maillard process resulted in unsatisfactory coloring of the product. Furthermore, the Maillard reaction produces harmful by-products such as acrylamide at high temperatures [7]. These defects limit the application of the Maillard reaction. Protein hydrolysates and polysaccharides can bind through other covalent interactions. Under the catalysis of transglutaminase (TGase), glycation of amino sugars (acyl receptors) can be achieved through the binding of glucosamine and oligochitosan –NH_2_ with protein Gln residues (acyl donors) [8]. Oligochitosan has various biological activities, such as antibacterial activity, antiviral activity, antioxidant activity, and high biocompatibility [9]. The primary amine properties of oligochitosan determine that it can serve as substrates for TGase catalytic glycation modification. Previous studies have shown that TGase-type glycation enhances the functional characterizations of proteins. The glycation reaction between oligochitosan and whey protein isolate catalyzed by TGase effectively improved the stability and antioxidant activity of the protein [10]. TGase induces the formation of glycated proteins from ovalbumin and glucosamine, thereby enhancing the functional properties of proteins such as solubility, emulsifying ability, and foaming ability [11].

Ultrasound, as an environmentally friendly and non-toxic physical modifier, have attracted widespread attention in food science. Ultrasonic treatment generates high-frequency vibrations through the cavitation effect, leading to the formation, growth, and collapse of microbubbles, resulting in local high temperature and high pressures. The cavitation effect caused by ultrasound can provide strong mechanical force, leading to peptide bond breaking and protein unfolding [12]. Therefore, it could alter the molecular characteristics of proteins to enhance their structure and functional properties. Meanwhile, ultrasound generates macroscopic vortices in the liquids, facilitating the mixing of reactants [13]. Ultrasound treatment accelerates the process of protein-polysaccharide conjugation and increases the chance of collisions between reactive groups. The cavitation effect generates free radicals, triggering oxidation, degradation, or cross-linking reactions. Therefore, the combination of enzymatic glycation and ultrasound treatment may have a better effect on improving the structure and functional properties of casein hydrolysate. In addition, ultrasound treatment can accelerate reaction kinetics, reduce reagent dependence, and simplify the process flows [14]. The combination of ultrasound treatment and enzymatic glycation can effectively reduce overall costs and enhance energy efficiency.

By combining TGase-type glycation and ultrasound treatment, this study proposes to improve the structure and functional properties of casein hydrolysate (CH). Casein was hydrolyzed by trypsin to generate CH. Under the TGase catalysis, CH and oligochitosan was used to prepared glycated CH, and the complex was treated with ultrasound to further enhance the degree of glycation. The structure, morphology, and functional characteristics of glycated CH product were evaluated. The effects of glycation and ultrasound on the foaming, antioxidant properties and solubility of CH were assessed. These findings provide new insights into TGase-type glycation combined with ultrasound treatment to further improve the functional properties and structural of proteins and expand their applications in the food industry.

## 2 Materials and methods

### 2.1 Materials

Casein was purchased from Sigma-Aldrich Co., Ltd. (Saint Louis, MO, USA). Oligochitosan was purchased from Golden-Shell Co. (Hangzhou, Zhejiang, China). Trypsin was purchased from Yuanye Biological Technology Co., Ltd. (Shanghai, China). TGase (enzyme activity of 147 units/g) was purchased from Jiangsu Yiming Fine Chemical Co., Ltd. (Jiangsu, China). Furthermore, all reagents were analytical reagent grade in the experiments.

### 2.2 Preparation of CH and glycated CH

The casein solution (pH 7.0) was supplemented with trypsin at a 5 % (w/w) ratio. After 3 h of hydrolysis at 50 °C, the solution was treated for 5 min at 100 °C to deactivate the enzyme. In order to obtain CH, the solution was centrifuged for 10 min at 4000 rpm, and the supernatant was collected. The DH of CH was 8.71 %. The method for preparing glycated CH was based on a previous study [15]. Oligochitosan (pH 7.5) was added to the CH solution (80 g/L, pH 7.5) to receive the molar ratio of acyl donor to acyl acceptor is 1:3. TGase was added to the solution. After 3 h of glycation reaction at 37 °C, the mixture was heated to 85°C for 5 min to deactivate the enzymes. The calculated amount of glycosyl was 87.14 g/kg protein. The sample solution (4 g/L) was treated by ultrasound. The parameter conditions of the ultrasound device were set as follows: power of 200 W, frequency of 20 kHz, time of 6 min, pulse duration of 3 s/3 s (on/off). During ultrasound treatment, the temperature was controlled to be ≤ 35 °C [16]. The ultrasound solution was recorded as CH (US), crosslinked CH (US) and glycated CH (US), respectively.

### 2.3 Physicochemical characterizations

#### 2.3.1 The particle size and zeta potential determination

Zeta Sizer Nano ZS-90 instrument (Malvern Instruments Ltd., Malvern, UK) was used to measure the particle size of samples, polydispersity index (PDI), and Zeta potential. The sample was dispersed in deionized water at 25 °C.

#### 2.3.2 Free amino groups determination

The method of o-phthalaldehyde (OPA) was employed to ascertain the free amino groups of the samples [17]. 3 mL of OPA reagent was added to a diluted sample solution (0.5 mg/mL, 100 µL) and stored at room temperature for 2 min away from light. The absorbance was recorded at a wavelength of 340 nm using an ultraviolet spectrophotometer. Leucine was the standard solution.

#### 2.3.3 Surface hydrophobicity determination

The method was conducted based on a previous study [18]. The 5 mL of sample solution with different concentrations was mixed with 20 μL of ANS (1-aniline-8-naphthalene sulfonate, 8.0 mmol/L) and the combination was permitted to react for 15 min in the dark. The fluorescence spectrophotometer was used to assess the relative fluorescence intensity of samples. The initial slope of fluorescence intensity and sample concentrations was used to calculate the surface hydrophobicity.

### 2.4 Structural characterizations

#### 2.4.1 UV spectral

The instrument was UV spectrophotometer (UV-2600, Shimadzu, Japan). Some modifications were based on a previous study [19]. The sample was completely dissolved in deionized water and diluted to 1 mg/mL concentration. The wavelength range of 200–450 nm was used to measure the absorbance of sample.

#### 2.4.2 Fluorescence spectroscopy

The fluorescence spectrophotometer (F-7100, Hitachi, Kyoto, Japan) was used to analyze the fluorescence spectra of samples. The sample was diluted to the concentration of 0.1 mg/mL after being dissolved in deionized water. The excitation wavelength was set to 280 nm, the operating voltage was 400 V, and the fluorescence spectrum scanning wavelength range was 300–450 nm [20].

#### 2.4.3 Fourier transform infrared (FTIR) spectroscopy

The method was conducted based on a previous study [21]. The 1:100 ratio was used to dispersed the sample in potassium bromide and subsequently ground. The transparent sheet was created by pressing the resultant mixture. Measurements of the infrared spectra were made over the wavenumber range of 4000 to 400 cm^−1^.

#### 2.4.4 Scanning electron microscopy (SEM)

The sample powder was pasted on a thin layer of aluminum, and then all the sample surfaces were coated with a thin layer of gold by a vacuum deposition process. The microstructure of sample was examined by SEM (SU8010, Hitachi, Tokyo, Japan) at different magnifications and the acceleration voltage was 5 kV.

#### 2.4.5 Transmission electron microscopy (TEM)

The TEM analysis of the samples was some modifications of previous study [22]. The microstructure of sample was examined by TEM (H-7650, Hitachi, Japan). The sample (0.1 mg/mL) was observed under a microscope at 80 kV.

### 2.5 Functional characteristics

#### 2.5.1 Solubility determination

The sample (1 mg/mL) was centrifuged at 12,000 rpm for 20 min. By measuring absorbance at 280 nm, the amount of protein in the supernatant was ascertained. The following formula was used to calculate protein solubility:

(1) $$Solubility\;\left(\%\right)\;=\frac{C_{1}}{C_{0}}\times 100\%$$

Where *C_1_* refers to the protein concentration in the supernatant, *C_0_* refers to the protein concentration in the stock solution.

#### 2.5.2 Foaming properties determination

The determination methods of Foaming capacity (FC) and stability (FS) have been modified according to the previous study [23]. The sample solution (1 mg/mL) was homogenized at 10,000 rpm for 2 min. The FC and FS were calculated as the percentage increase in the volume of protein solution following homogenization and the percentage of foam that remained after 30 min, respectively.

#### 2.5.3 Emulsifying properties determination

The determination methods of Emulsifying activity index (EAI) and emulsion stability index (ESI) have been modified according to the previous study [24]. 3 mL of soybean oil were combined with 9 mL of protein solution (1 mg/mL), and the mixture was homogenized for 2 min at 10,000 rpm. 10 mL of SDS solution (1 mg/mL) was used to dilute the 100 μL of emulsion from the bottom of container. The absorbance values at 500 nm for 0 min and 10 min were measured. The following formula was used to calculate EAI and ESI:

(2) $$\mathrm{EAI}\left(m^{2}/g\right)=\frac{2\;\times \;2.303\;\times \;A_{0}\;\times \;D}{C\times \varphi \times \theta \times 10000}$$

(3) $$\mathrm{ESI}\left(\%\right)=\frac{A_{10}}{A_{0}}\times 100$$

Where *A_0_* and *A_10_* represent the absorbance values obtained at 0 and 10 min, D represents the dilution ratio, C represents the concentration of sample, φ represents the oil volume fraction, θ represents the optical path length (1 cm).

#### 2.5.4 DPPH radical scavenging activity determination

After 1 mL of DPPH solution mixed with 2 mL of sample solution, the dark reaction was carried out for 30 min. The ultraviolet spectrophotometer was used to measure the absorbance of mixture at 517 nm. The following formula was used to calculate DPPH radical scavenging rate:

(4) $$DPPHradicalscavengingrate\left(\%\right)=\left(1-\frac{A_{2}-A_{1}}{A_{0}}\right)\times 100\%$$

Where *A_0_* is the absorbance of anhydrous ethanol mixed with DPPH solution, *A_1_* is the absorbance of the sample mixed with anhydrous ethanol, *A_2_* is the absorbance of the sample mixed with DPPH solution.

#### 2.5.5 Differential scanning calorimetry (DSC)

DSC (DSC250, TA, USA) was used to analyze the thermal characteristics of the sample. The tablet press was used to compact the 5 mg sample after it had been put in an aluminum pan. In the nitrogen environment, the temperature rise range was 30–180 °C. The changes of sample chromatogram were analyzed.

### 2.6 Statistical analysis

Each test was repeated three times, and the experimental results are shown as the mean ± standard deviation. One-way ANOVA and the SPSS 25.0 statistical analysis program were employed and significant differences were determined between tests (*p* < 0.05). Excel 2021 and Origin 2021 software were used in chart generation.

## 3 Results and discussion

### 3.1 Physicochemical characterization

#### 3.1.1 The particle size and zeta potential analysis

**Fig. 1:**
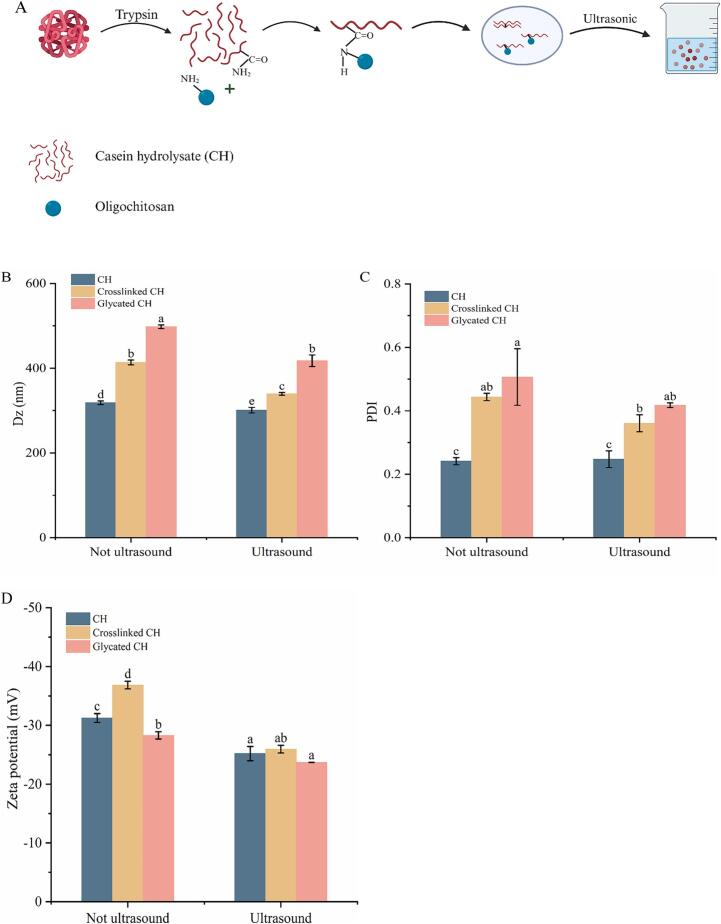
Schematic illustration (A) of the preparation of glycated CH. Created in BioRender. Particle size (B), polydispersity index (C) and zeta-potential (D) of CH, crosslinked CH and glycated CH before and after ultrasound treatment. Difference lowercase letters indicate a significant difference at *p* < 0.05.

The preparation of samples was shown in Fig. 1A. The average particle size, PDI and zeta-potential of CH, crosslinked CH and glycated CH before and after ultrasound treatment were shown in Fig. 1B-D. The particle size of crosslinked CH was greater than CH, which might be due to the crosslinked reaction induced the formation of more macromolecular polymers. This result aligned with the findings published by Sun et al. [25].The addition of oligochitosan increased the particle size of CH, which could be ascribed to the formation of the glycated CH by crosslinking of TGase, and the introduction of sugar chains might affect the particle size of the complexes [26]. The particle size of CH (US), crosslinked CH (US) and glycated CH (US) decreased to 300.90 ± 6.57 nm, 339.60 ± 3.21 nm, and 417.60 ± 13.60 nm, respectively. The high-frequency vibrations and cavitation effects of ultrasound can disrupt the aggregates of proteins or macromolecular structures, resulting in the more uniform and regular morphology of the complex and smaller sample particle size [27]. Ultrasound treatment can effectively improve the stability of the complex. The zeta potential of the glycated CH was inferior to that of CH, which may be due to the greater separation between the surface charge of the protein and the continuous phase following oligochitosan grafting. It may also be due to the oligochitosan-grafted protein shielding the surface charge of CH, resulting in a further decrease in charge [28]. Following ultrasound treatment, the absolute value of the zeta potential decreased. This due to cavitation effect of ultrasound can unfold CH structure and disrupt protein aggregates, thereby changing net charge of CH surface. In addition, the PDI of samples remained small, indicating an excellent dispersion and homogeneity of the sample system.

#### 3.1.2 Free amino groups analysis

**Fig. 2:**
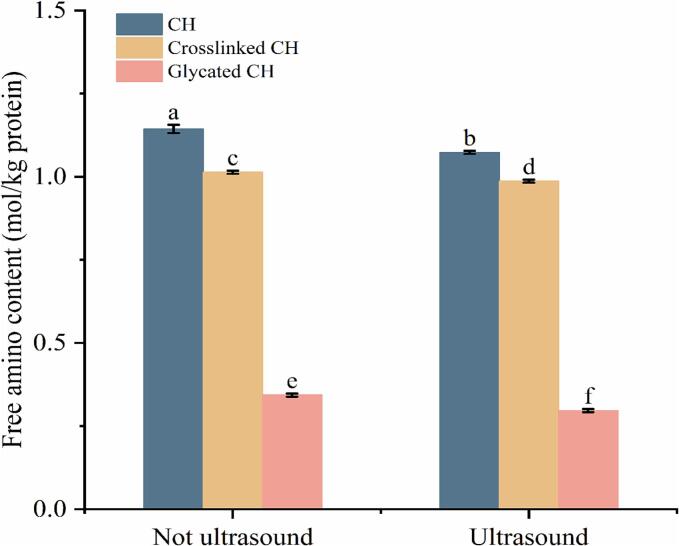
The free amino groups of CH, crosslinked CH and glycated CH before and after ultrasound treatment. Difference lowercase letters indicate a significant difference at *p* < 0.05.

The change of free amino content in protein can be used to reflect the crosslinked and glycation degree of protein [29]. Fig. 2 exhibits that the free amino content of crosslinked CH decreased from 1.14 ± 0.01 mol/kg protein to 1.01 ± 0.01 mol/kg protein, and that of glycated CH decreased to 0.34 ± 0.01 mol/kg protein. This due to TGase catalyzed the crosslinked reaction between glutamine and lysine residues [30]. In the process of glycation, CH and oligochitosan form a new ε- (γ-glutamine)-lysine peptide bond under the action of TGase, resulting in the reduction of free amino content. After ultrasound treatment, the content of free amino decreased, which may be due to the unfolding of proteins caused by ultrasound. Through the implosion of microbubbles generates localized shear forces, turbulence, and mixing, the degree of glycation was enhanced, and the secondary and tertiary structures of proteins were destroyed, causing them to unfold from a tightly folded state and expose the originally free amino.

#### 3.1.3 Surface hydrophobicity analysis

**Fig. 3:**
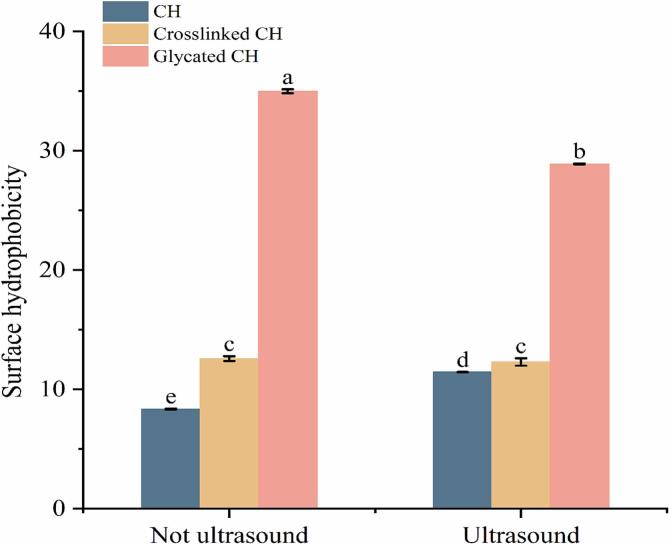
The surface hydrophobicity of CH, crosslinked CH and glycated CH before and after ultrasound treatment. Difference lowercase letters indicate a significant difference at *p* < 0.05.

The surface hydrophobicity changes of CH, crosslinked CH, glycated CH are shown in Fig. 3. The protein is usually modified to change its structure, which can expose more hydrophobic regions [31]. Crosslinked CH has better surface hydrophobicity than CH because TGase altered the structure of protein and revealed more hydrophobic groups. Notably, glycated CH showed the highest surface hydrophobicity, indicating that glycation can significantly improve the surface hydrophobicity of CH (*p* < 0.05). Glycation reaction increased the intermolecular electrostatic repulsion of CH and changed structure of the protein, thus exposing the hydrophobic amino acids embedded on the surface [32]. This result was consistent with the previous study that glucans improved the surface hydrophobicity of ovalbumin [33]. After ultrasound treatment, the surface hydrophobicity of proteins decreases due to cavitation, which exposes more hydrophilic groups embedded in the proteins on the surface, thereby reducing surface hydrophobicity. The formation of soluble protein aggregates may be another factor due to hydrophobic interactions between exposed hydrophobic amino acid residues [34].

### 3.2 Structural characterizations

#### 3.2.1 UV spectral analysis

**Fig. 4:**
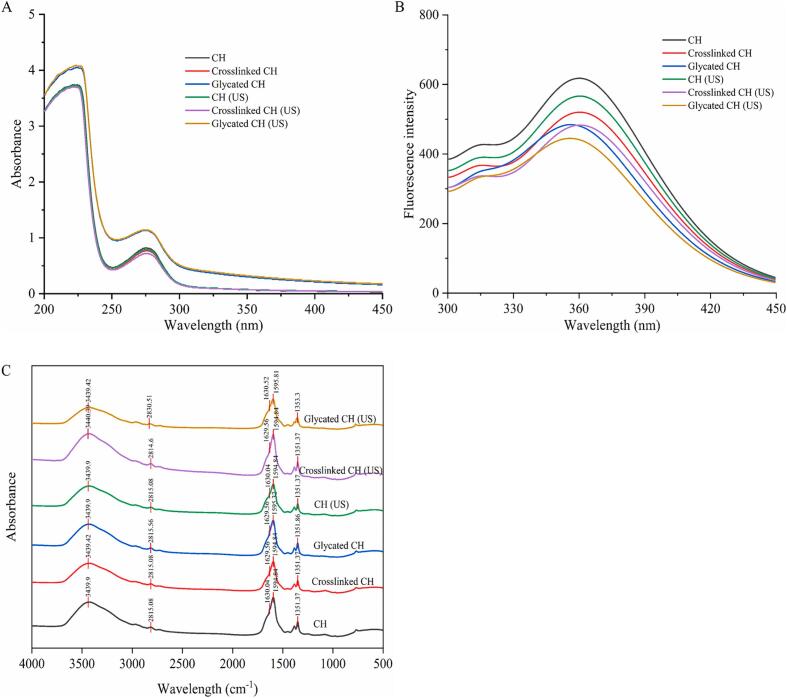
The ultraviolet absorption spectrum (A), fluorescence spectroscopy (B) and FTIR (C) of CH, crosslinked CH and glycated CH before and after ultrasound treatment. Difference lowercase letters indicate a significant difference at *p* < 0.05.

During the formation or structural changes of protein complexes, significant changes occur in UV absorbance spectra [35]. As shown in Fig. 4A, there are two obvious characteristic absorption peaks in the ultraviolet spectrum, located at 220–225 nm and 272–277 nm, respectively. The wavelength range of the former was primarily characterized by changes in peptide bonds, while the wavelength range of the latter was defined by the characteristic absorption peaks of conjugated double bonds of tryptophan and tyrosine residues. The intensity of the glycated CH peptide bond absorption peak increased and undergone a red shift. This may be due to the internal stretching of polysaccharide and protein molecules, which exposed the chromophore groups on the surface of CH and increased the absorption intensity [36]. This result was consistent with the research findings by Feng et al. [37]. In addition, ultrasound treatment had no significant impact on the ultraviolet absorption of the samples.

#### 3.2.2 Fluorescence spectroscopy analysis

The interaction between proteins and ligands can be detected by fluorescence quenching [38]. Proteins contain tryptophan and tyrosine residues, which can produce endogenous fluorescence at specific excitation wavelengths [39], making them highly sensitive to spatial and structural changes in proteins [40]. As shown in Fig. 4B, compared with CH, the fluorescence intensity of crosslinked CH and glycated CH was significantly reduced. This may be due to the formation of aggregates under the effect of TGase, resulting in fluorescence shielding, or the formation of the covalent bond between the ε-amino group of lysine in CH and the γ-hydroxyamino group of glutamic acid. This indicated a strong covalent interaction between CH and oligochitosan. This result was consistent with the research that showed a decrease in protein fluorescence intensity after the addition of TGase [41]. The maximum fluorescence of glycated CH showed a slight blue shift, indicating that the glycation of CH resulted in structure changes of proteins. The fluorescence intensity of CH significantly decreased after ultrasound treatment. This may be due to the cavitation effect of ultrasound treatment, which unfolded the structure of CH and exposed more reactive groups, thereby enhancing glycation reactions. This further confirmed that ultrasound treatment can effectively improve the degree of glycation to a certain extent.

#### 3.2.3 FTIR analysis

As shown in Fig. 4C, due to the stretching vibration of hydrophilic O-H groups, CH had a wide absorption band at 3700–3000 cm^−1^ [42]. The peak at 2815.08 cm^−1^ corresponded to hydrophobic C-H stretching and bending vibrations. Absorption peaks characterized by amide bonds were observed at 1630.04 cm^−1^ (amide I, C-O bonds), 1594.84 cm^−1^ (amide II, N-H bonds), and 1351.37 cm^−1^ (amide III, C-N and N-H bonds) [43], [44]. The blue shift of glycated CH in amide I indicated the involvement of C-O bonds in the reaction. However, glycated CH (US) exhibited a red shift, indicating that ultrasound treatment further affected the protein secondary structure of glycated CH (US). This may be related to the unfolding of protein molecules caused by ultrasound cavitation. Ultrasound treatment promoted the production of glycation products and changed in protein structure [45].

#### 3.2.4 SEM analysis

**Fig. 5:**
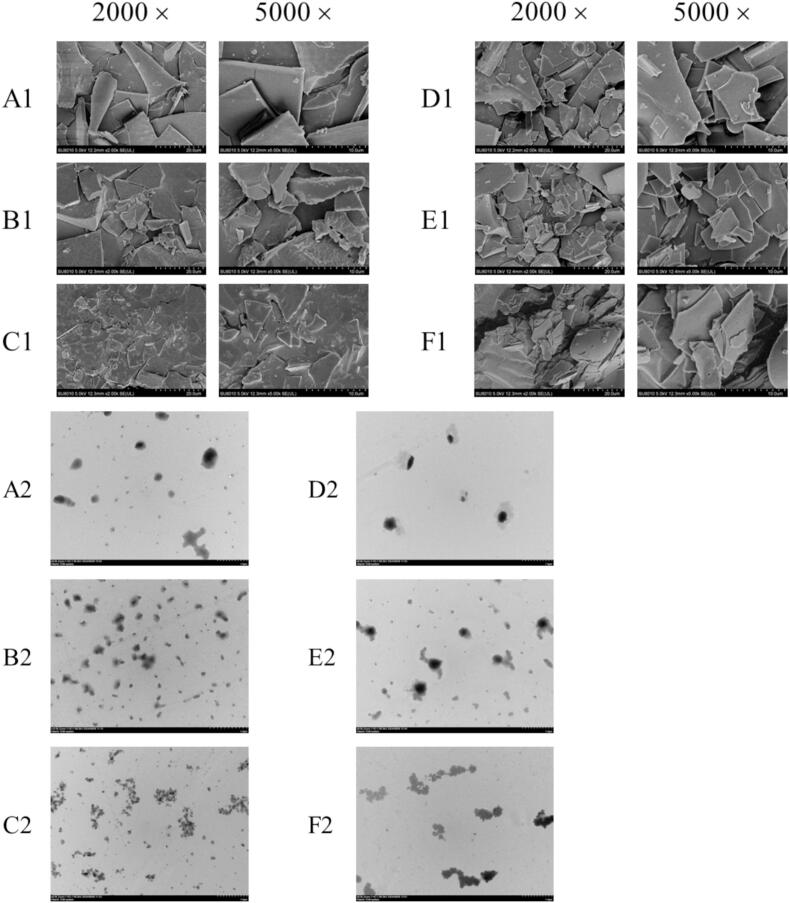
SEM (A1-F1) and TEM (A2-F2) images of CH, crosslinked CH and glycated CH before and after ultrasound treatment. Difference lowercase letters indicate a significant difference at *p* < 0.05.

As shown in Fig. 5A1-F1, the micromorphology of CH, crosslinked CH and glycated CH before and after ultrasound were analyzed by SEM at 2000 × and 5000 × . CH exhibited an irregular shape and a smooth surface. The structure of CH was changed by crosslinking of TGase, resulting in a rougher surface of the crosslinked CH. This may be because crosslinked CH has stronger hydrophilic properties than CH, resulting in more stable adsorption of water droplets and crosslinked CH. However, during the freeze-drying process, proteins lose their surface moisture, resulting in this prominent state. Compared with crosslinked CH, glycated CH had a looser structure, indicating significantly changed in protein structure. After ultrasound treatment, the degree of protein fragmentation increased. The high-pressure microjets and shock waves generated by bubble collapse in cavitation can tear apart the secondary and tertiary structures of proteins, leading to the unfolding or breaking of protein molecules. CH (US), crosslinked CH (US), and glycated CH (US) demonstrated excellent dispersion. This result was similar to the previous study [46]. This was because ultrasound created a core–shell structure in the particles, disrupting the interactions between molecules and making them more independent. Therefore, ultrasound can effectively improve the dispersion of CH.

#### 3.2.5 TEM analysis

The TEM images of the samples before and after ultrasound were presented in Fig. 5A2-F2. It can be seen that CH is spheroidal shape. The particle size of crosslinked CH was large than that of CH. This was attributed to TGase catalyzing acyl transfer between lysine and glutamine residues, leading to CH aggregation due to crosslinking. The TEM image of glycated CH showed significant changes, indicating that glycation occurred and affected the morphological structure. Furthermore, the particle sizes of CH, crosslinked CH and glycated CH gradually increases, which was consistent with the above particle size results. Simultaneously, a decrease in particle size was observed for CH (US), crosslinked CH (US) and glycated CH (US). The result was consistent with the research findings that ultrasound treatment could reduce proteins particle size [47].

### 3.3 Functional characteristics

#### 3.3.1 Solubility analysis

**Fig. 6:**
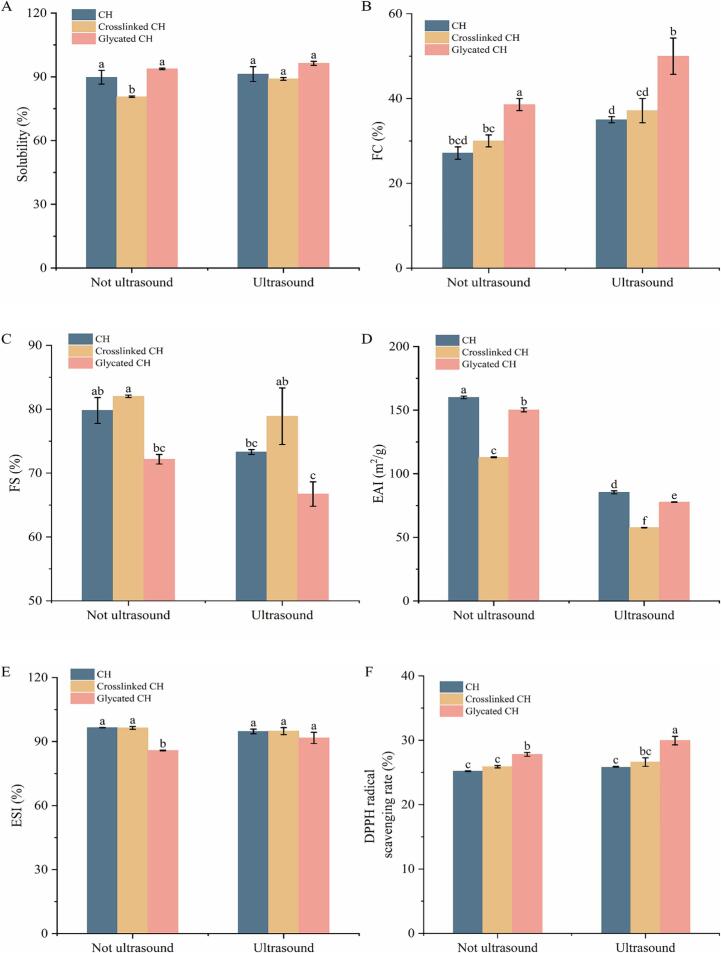

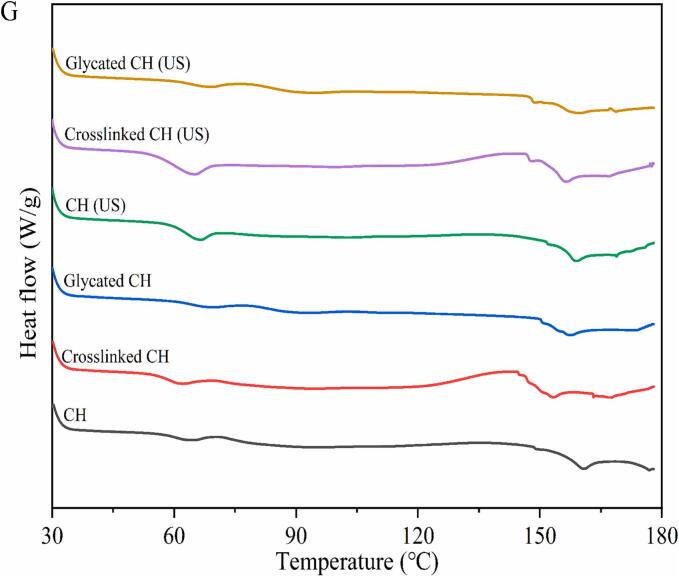
The solubility (A) of CH, crosslinked CH and glycated CH before and after ultrasound treatment. The FC (B) and FS (C) of CH, crosslinked CH and glycated CH before and after ultrasound treatment. The EAI (D) and ESI (E) of CH, crosslinked CH and glycated CH before and after ultrasound treatment. DPPH radical scavenging activity (F) of CH, crosslinked CH and glycated CH before and after ultrasound treatment. The DSC (G) of CH, crosslinked CH and glycated CH before and after ultrasound treatment. Difference lowercase letters indicate a significant difference at *p* < 0.05.

Solubility is an important indicator for evaluating protein aggregation behavior. Proteins with excellent solubility can be more evenly and quickly dispersed in the system, which helped to disperse proteins to the oil–water interface and enhance other functional characteristics. As shown in Fig. 6A, compared with CH, the solubility of crosslinked CH decreased from 89.80 ± 3.23 % to 80.64 ± 0.35 %, and the solubility of glycated CH reached 93.75 ± 0.36 %. The decrease in solubility of crosslinked CH may be due to the formation of crosslinked between the glutamyl residue and the ε-amino group of lysine, resulting in the formation of macromolecular polymers. This was consistent with the results that TGase reducing the solubility of soy protein isolate [48]. The reason for the increase in solubility of glycated CH was the introduction of hydrophilic oligochitosan, which increased the number of hydrophilic hydroxyl groups. This improved the binding between proteins and water, and created steric hindrance on the protein surface, hindering protein polymerization [49]. Compared with samples without ultrasound treatment, the solubility of CH (US), crosslinked CH (US) and glycated CH (US) all increased. This is because cavitation destroyed insoluble protein aggregates and reassembled them into soluble aggregates [50]. The result was consistent with the study that ultrasound can increase the solubility of canola protein [51]. Therefore, ultrasound combined with glycation may be an efficient way to increase the solubility of CH.

#### 3.3.2 Foaming properties analysis

Proteins are composed of hydrophobic and hydrophilic amino acids, with a typical amphiphilic structure and excellent surface activity. During the stirring process, it tended to form bubbles at the air–water interface. As shown in Fig. 6B-C, the FC of glycated CH increased from 27.15 ± 1.44 % to 38.57 ± 1.43 %. The FC of glycated CH increased, which may be due to the greater solubility of oligochitosan and its ability to transfer to the molecular interface faster than proteins, thereby increasing the surface activity of CH [52]. This indicated that the glycation reaction between CH and oligochitosan could increase the surface activity of proteins. The FC of glycated CH (US) further increased. This may be because the increase in solubility facilitated the rapid diffusion, adsorption, stretching and rearrangement of proteins at the gas–liquid interface, thereby increasing the foaming capacity [53]. However, the FS of glycated CH decreased, which may be due to the increase in particle size and electrostatic repulsion between molecules, limiting its rapid adsorption at the air–water interface [54]. After ultrasound treatment, the FS of glycated CH (US) decreased. This may be because the high temperature generated by ultrasound may destroy covalent and non-covalent bonds, resulting in the breakdown of protein aggregates, which is not conducive to the foam stability of proteins.

#### 3.3.3 Emulsifying properties analysis

The EAI and ESI of CH, crosslinked CH and glycated CH before and after ultrasound treatment were shown in Fig. 6D-E. Compared with CH, the EAI of crosslinked CH decreased from 160.02 ± 1.02 m^2^/g to 112.94 ± 0.37 m^2^/g, and the EAI of glycated CH was 150.25 ± 1.57 m^2^/g. The EAI of glycated CH decreased significantly (*p* < 0.05), because the polymers hindered the interfacial absorption of proteins and promoted the aggregation of proteins in the interface area of emulsion [55]. The EAI of CH (US), crosslinked CH (US), and glycated CH (US) decreased, which may be due to the fact that ultrasound treatment can break the non-covalent bonds that preserved the spatial protein structure and caused protein aggregation, thereby reducing the adsorption efficiency of proteins at the oil–water interface. The ESI of the samples did not show significant changes.

#### 3.3.4 DPPH radical scavenging activity analysis

The antioxidant activity studied in proteins depends on their composition and structural characteristics [56]. As can be seen from Fig. 6F, the DPPH radical scavenging rates of CH, crosslinked CH and glycated CH were 25.18 ± 0.06 %, 25.90 ± 0.19 %, and 27.81 ± 0.3 %, respectively., The crosslinked CH mediated by TGase slightly increased the content of hydrophobic amino acids, and most hydroxyl groups contain active hydrogen [57]. Notably, the DPPH radical scavenging rate of glycated CH was the highest, because the oligochitosan molecular chain contains large numbers of hydroxyl and amino groups, which may bind with free radicals to achieve the purpose of scavenging free radicals. The DPPH radical scavenging rate of samples after ultrasound treatment is generally higher. This may be due to the collapse of bubbles forms shear forces and shock waves carrying a large quantity of energy, which destroyed the secondary and tertiary structures of proteins, exposing the antioxidant active groups originally buried inside, as the ultrasound effect may alter the spatial structure of the sample, thereby enhancing its radical scavenging rate. This confirmed that the antioxidant activity of CH was increased by TGase-type glycation and ultrasound treatment.

#### 3.3.5 DSC analysis

Thermal stability is crucial for proteins in the food industry [58]. As shown in Fig. 6G, the thermal deformation of CH had two endothermic peak temperatures: 62.96 °C and 159.8 °C. Compared with CH, the first peak denaturation temperature of crosslinked CH decreased by 2.08 % from 62.96 °C to 61.65 °C, and the second peak denaturation temperature decreased by 3.69 % from 159.8 °C to 153.91 °C. This indicated that the reaction of TGase slightly reduced the thermal stability of CH. After glycation, compared with CH, the temperature of the first denaturation peak of glycated CH increased from 62.96 °C to 69.56 °C, an increase of 10.48 %. The temperature of the second denaturation peak decreased from 159.8 °C to 158.66 °C, a decrease of 0.71 %. Compared with crosslinked CH, the first peak denaturation temperature of glycated CH increased by 12.83 %, and the second peak denaturation temperature increased by 3.09 %. This phenomenon indicated that the addition of oligochitosan improved the thermal stability of CH, which may be due to the covalent interaction between polysaccharide and protein enhancing thermal stability. After ultrasound treatment, the two peak denaturation temperatures of CH (US), crosslinked CH (US) and glycated CH (US) increased, indicating that ultrasound treatment can appropriately improve the thermal stability of proteins. This may be due to appropriate ultrasound treatment promoting glycation reactions and inhibiting crosslinking reactions between proteins [59]. The results showed that ultrasound combined with glycation could improve the denaturation temperature and thermal stability of protein.

## 4 Conclusion

In summary, ultrasound treatment could promote the degree of TGase-type glycation. This process decreased the content of free amino and fluorescence intensity in the protein system. During the formation or structural changes of glycated protein complexes, the UV absorption spectrum undergone significant changes, while ultrasound treatment had no significant effect on the UV absorption of glycated proteins. The combination of TGase-type glycation and ultrasound treatment exhibited smaller particle size, uniform distribution, and better surface hydrophobicity of CH. The spatial structure of modified CH changed after ultrasound treatment. Moreover, the foaming capacity, solubility, antioxidant capacity and thermal stability of CH also were improved. The results indicated that the combination of ultrasound treatment and enzymatic glycation provides an efficient and safe protein modification solution for the food industry through the synergistic effects of physical modification and biocatalysis, which is helpful for the development of functional food and pharmaceutical health products.

## CRediT authorship contribution statement

**Huimin Wang:** Writing – original draft, Investigation. **Yujun Jiang:** Supervision, Resources. **Jia Shi:** Writing – review & editing, Supervision.

## Declaration of competing interest

The authors declare that they have no known competing financial interests or personal relationships that could have appeared to influence the work reported in this paper.
